# Exploring the Escalating Trends and Variances in Attention Deficit Hyperactivity Disorder Prevalence: A Critical Review

**DOI:** 10.7759/cureus.92423

**Published:** 2025-09-16

**Authors:** Taha Hussain, Prashant Neupane, Vineet Khunger

**Affiliations:** 1 Faculty of Biology, Medicine and Health, University of Manchester, Manchester, GBR; 2 Department of Vascular Surgery, Manchester Royal Infirmary, Manchester, GBR; 3 General Practice, Haughton Thornley Medical Centres, Hyde, GBR

**Keywords:** attention deficit hyperactive disorder (a.d.h.d.), attention deficit hyperactivity disorder (adhd), clinical psychiatry, general & adult psychiatry, lower socioeconomic group, neurology, public health and adhd

## Abstract

The objective of this review is to present the prevalence of ADHD (Attention Deficit Hyperactivity Disorder) from both a global perspective and across various subgroups of people, using evidence from the current literature. This review will also explore variation in ADHD prevalence based on various factors such as race and gender. The review aims to provide insights into potential mechanisms that explain the synthesised data.

We conducted a comprehensive electronic literature search on PubMed, Web of Science, and Google Scholar using search terms such as "ADHD prevalence" and "Epidemiology of ADHD." Papers were initially screened by abstract for relevance before undergoing a full-text assessment to ensure satisfaction of the inclusion criteria. Papers were rated based on their methodological quality using a pre-determined criterion.

The prevalence of ADHD diagnoses has been increasing significantly over the last three decades. Subgroup analysis showed that the prevalence of diagnosed ADHD was significantly lower in girls, ethnic minorities, and underprivileged children. A similar increase in ADHD symptoms does not accompany the increase in ADHD diagnoses. Instead, increased awareness among underrepresented groups is a key driving factor. When study methods were controlled for, the year of publication had no significant effect on the reported prevalence. There is no significant geographic variation in ADHD prevalence, as variation among studies can be largely attributed to methodological inconsistencies.

The study shows the importance of increasing consistency in ADHD diagnostic methods to reduce potential over- and underdiagnosis of ADHD. The study also highlights the need for interventions to reduce barriers faced by underrepresented groups in receiving ADHD diagnoses.

## Introduction and background

ADHD (Attention Deficit Hyperactivity Disorder) is a chronic psychiatric disorder that can have debilitating effects on an individual’s functioning, most commonly in children. More specifically, children with ADHD exhibit three main groups of symptoms, which are inappropriate levels of hyperactivity, impulsivity (acting without thinking, interrupting, and strong emotional reactions), and inattentiveness for their level of development. This led to ADHD being divided into its three subtypes based on the predominant type of symptom, which includes ADHD-inattentive (difficulty focusing, organising, and avoiding distractions), ADHD-hyperactive (restlessness and fidgeting), and ADHD-combined [1]. It is found that the inattentive subtype is prevalent in about 18.3% of the total patients, while hyperactive/impulsive and combined represent 8.3% and 70%, respectively. The two formal diagnostic criteria for ADHD are DSM-5 (Diagnostic and Statistical Manual of Mental Disorders, 5th edition) and ICD-10 (International Classification of Diseases), although there is inconsistency among the criteria and diagnostic practices [2-4]. ADHD can manifest throughout all stages of an individual's life. Symptoms typically start in early primary school years, when children experience academic difficulties and low self-esteem. At the age of 11, symptoms progress to disruptive behaviours, learning delays, and poor social skills with poor academic achievement [5]. In up to 65% of children, symptoms progress into adulthood, which leads to poor long-term outcomes, including employment dismissal, relationship failures, and substance abuse [6].

The objective of this paper is to understand and explain the increasing prevalence of ADHD and the variation of ADHD prevalence amongst different demographic groups by critically reviewing 13 selected studies. For example, many studies have repeatedly shown that ADHD has been less frequently diagnosed in girls, ethnic minorities, and children from lower socioeconomic status [7,8]. It is important to understand trends in ADHD prevalence as it provides an insight into the factors driving these trends, which enables public health institutions to enact necessary interventions to address these factors to relieve the health burden on individuals [9]. Furthermore, there is significant variation in ADHD both nationally and internationally; hence, understanding patterns of variation may highlight disparities that need to be addressed [10]. For example, in the past, ADHD has been diagnosed disproportionally less in girls compared to boys, whilst experiencing greater levels of perceived stress [11].

Despite variation in reported ADHD prevalence figures, current data show that ADHD prevalence has been increasing over the last three decades [12]. However, evidence also shows that there is no corresponding similar increase in ADHD symptoms [13], which suggests that the increasing prevalence is due to external factors such as improved awareness of ADHD among underrepresented groups and inclusive changes to diagnostic criteria [14]. For example, following a number of changes to diagnostic criteria which recognise a greater scope of symptoms and define disease characteristics, ADHD diagnosis rates between 1991 and 2008 increased by 5.6-fold for girls and only 3.7 for boys [15,16].

Furthermore, exploring disparities in ADHD prevalence helps answer the question of whether ADHD is overdiagnosed or underdiagnosed in certain groups of people, both of which can have a significant negative impact on those affected, as well as fiscal implications for healthcare providers responsible for treating ADHD. Overdiagnosis leads to unnecessary treatment with stimulant medications, which expose patients to long-term side effects such as improper growth and development [17]. Likewise, underdiagnosis can severely impact social functioning due to the disruptive symptoms of untreated ADHD [18]. Increasing ADHD prevalence poses challenges and limitations in pharmacological treatment, such as stimulant diversion and poor adherence, which will need to be addressed [19].

This critical review follows the IMRAD structure (introduction, methods, results, discussion, and conclusion). It aims to critically appraise 13 relevant, selected studies to provide insights into ADHD prevalence and implications for public health and clinical practice. 

The methods outline the search strategy and systematic approach utilised to gather the information for this review. In the results section, our goal is to clearly present and integrate findings from 13 studies; this encompasses numerical information regarding ADHD prevalence categorised by age, sex, race, ADHD subtype, and geographical area. The discussion section seeks to investigate the elements contributing to variations in ADHD prevalence and trends, as well as their potential future impacts from a public health viewpoint. The conclusion section seeks to wrap up the review by offering a brief summary that highlights the main findings of the assessment.

Evidence shows that methodological differences amongst studies account for a significant amount of variation in reported prevalence figures [13]. For example, the BEfragung zum seeLischen WohLbefinden und VerhAlten (BELLA; Survey on Mental Health of Children and Adolescents) study indicates that prevalence rates are lower when ICD-10 is used in comparison to DSM criteria because ICD-10 criteria are more restrictive [2]. This is because the ICD-10 criteria require symptoms of both inattention and hyperactivity/impulsivity, whereas DSM-5 allows for different subtypes of ADHD, whereby individuals can have domain-specific symptoms to meet the diagnostic criteria [2]. Furthermore, the majority of the studies either use interview-based methods or data from medical records to identify which participants have ADHD. Interviews (e.g., of parents/teachers) create subjectivity and introduce social biases in the study method. For example, some cultures, such as the West, are more tolerant of hyperactive and impulsive behaviours, which may lead to less perceived abnormality in a child’s behaviour [20]. Ultimately, a lack of uniformity in diagnostic and study methods decreases the comparability and generalizability of study results.

## Review

Methods

Inclusion Criteria

Papers that were published in 2000 or later were considered, given that the primary outcome was ADHD prevalence or variables influencing variation in ADHD prevalence. Studies with a sample size greater than 1000 were included. Longitudinal studies, cohort studies, systematic reviews, and meta-analyses were included in the inclusion criteria. Studies that utilised standardised methods to identify participants with ADHD were included. These methods included the use of well-established diagnostic criteria such as DSM or ICD-10; comprehensive assessments of participants; pre-made questionnaires based on DSM/ICD-10 criteria; and holistic assessment of participants using information from multiple sources, such as teacher-based reports and parent interviews. 

Exclusion Criteria

The following exclusion criteria were adopted: (i) studies published before 1996; (ii) studies which do not utilise standardised diagnostic methods (e.g., unvalidated self-reported symptoms); (iii) studies which do not report on ADHD prevalence, trends, variances or features. Only studies which provided an in-depth insight into procedural details were considered. Procedural details included methods, participant characteristics, and clear sources of data.

Search Strategy

A comprehensive electronic literature search was carried out on PubMed, Web of Science, and Google Scholar to identify relevant studies published from 2000-2024. Some of the key search terms used were "ADHD prevalence," "ADHD trends", "ADHD variation," and "epidemiology of ADHD." Initially, studies were screened by abstracts to identify potentially eligible studies, followed by a comprehensive full-text assessment to ensure fulfilment of the inclusion criteria.

Data Extraction

We selected a total of 13 studies which met the inclusion criteria. These studies were systematically assessed to extract the necessary information to complete the data extraction form. The data extracted from each study includes study design, publication year, first author, diagnostic method utilised (e.g., ICD or DSM), participant characteristics, reported ADHD prevalence rate(s), subgroup analyses and factors influencing prevalence. Data extraction was conducted by the first author and then reviewed again by the same author after a one-week interval to ensure accuracy and consistency.

Data Analysis

The selected studies were analysed to obtain reported prevalence rates, trends and variation, which were compared amongst studies to gain an insight into the heterogeneity and commonalities in the study findings. Descriptive statistics, including confidence intervals and odds ratios, were obtained and analysed to summarise the findings of each study. We examined the subgroup analysis for relevant studies to explore variation in ADHD prevalence across different sociodemographic characteristics such as race and gender.

An analysis was conducted to explore the temporal relations between prevalence trends and associated factors to provide mechanistic insights into the growing prevalence of ADHD diagnoses. 

Quality Assessment

Studies were assessed on their methodological quality using a predetermined criterion. The criteria considered various characteristics of the study, including sample size, diagnostic method accuracy, statistical precision, subgroup analyses, duration of study, and potential sources of bias. Each study was critically reviewed to identify its strengths and limitations to ensure that interpretations are considered in the context of the methodological background of the study. This ensures that the data compiled from the various studies is robust.

Data Synthesis

Information about each study from the data extraction form was aggregated to identify ADHD prevalence figures, trends, and variational patterns amongst demographic groups. Comparisons were made between studies using metrics such as confidence intervals and odds ratios to elucidate commonalities and differences between study findings. The key findings of the studies were summarised to provide a narrative review highlighting common themes and trends across studies, for example, racial and ethnic disparities in ADHD prevalence. The studies were also grouped based on common patterns and trends to provide insights into potential mechanisms explaining the study findings, whilst recognising and addressing inconsistencies in the collated data to ensure robustness of the synthesised evidence.

By incorporating a variety of studies in the data synthesis, this review presents a broad, global view of ADHD prevalence, as well as highlighting key differences between studies based on their individual characteristics, such as methodology and sample population. 

Results

Temporal Trends in ADHD Prevalence

**Figure 1:**
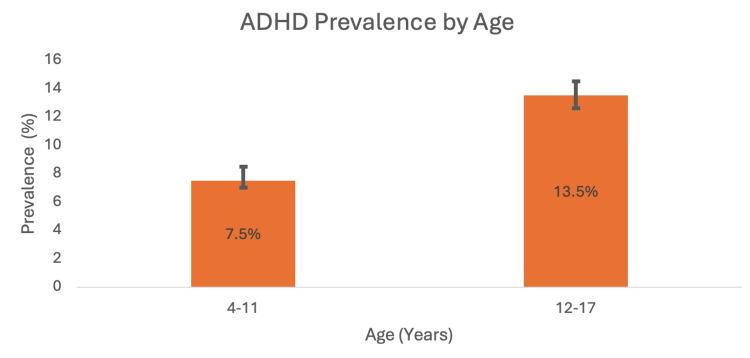
ADHD prevalence in children aged 4-11 years and 12-17 years with 95% confidence intervals (2015-2016) ADHD: attention deficit hyperactivity disorder Source: [12]

**Figure 2:**
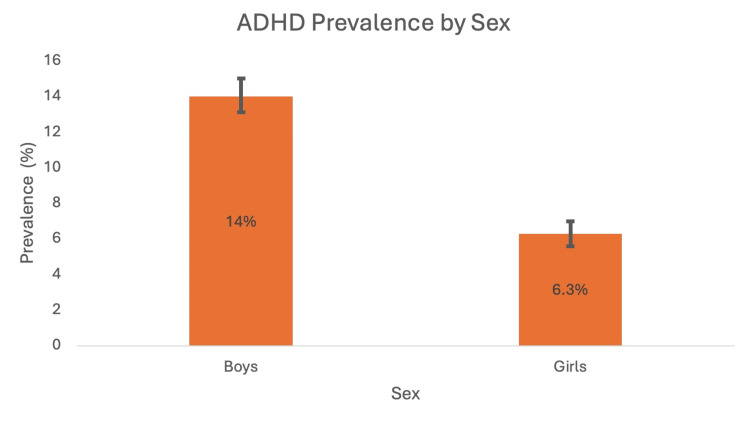
ADHD prevalence for boys and girls during 2015-2016 with 95% CI intervals ADHD: attention deficit hyperactivity disorder Source: [12]

**Figure 3:**
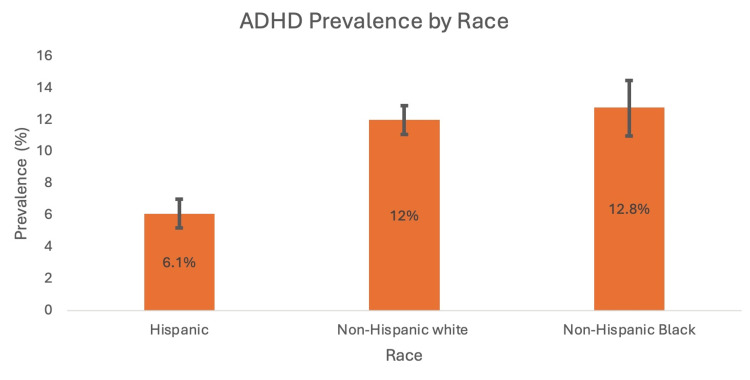
ADHD prevalence in Hispanic, non-Hispanic White and non-Hispanic Black people with 95% CI intervals (2015-2016) ADHD: attention deficit hyperactivity disorder Source: [12]

**Figure 4:**
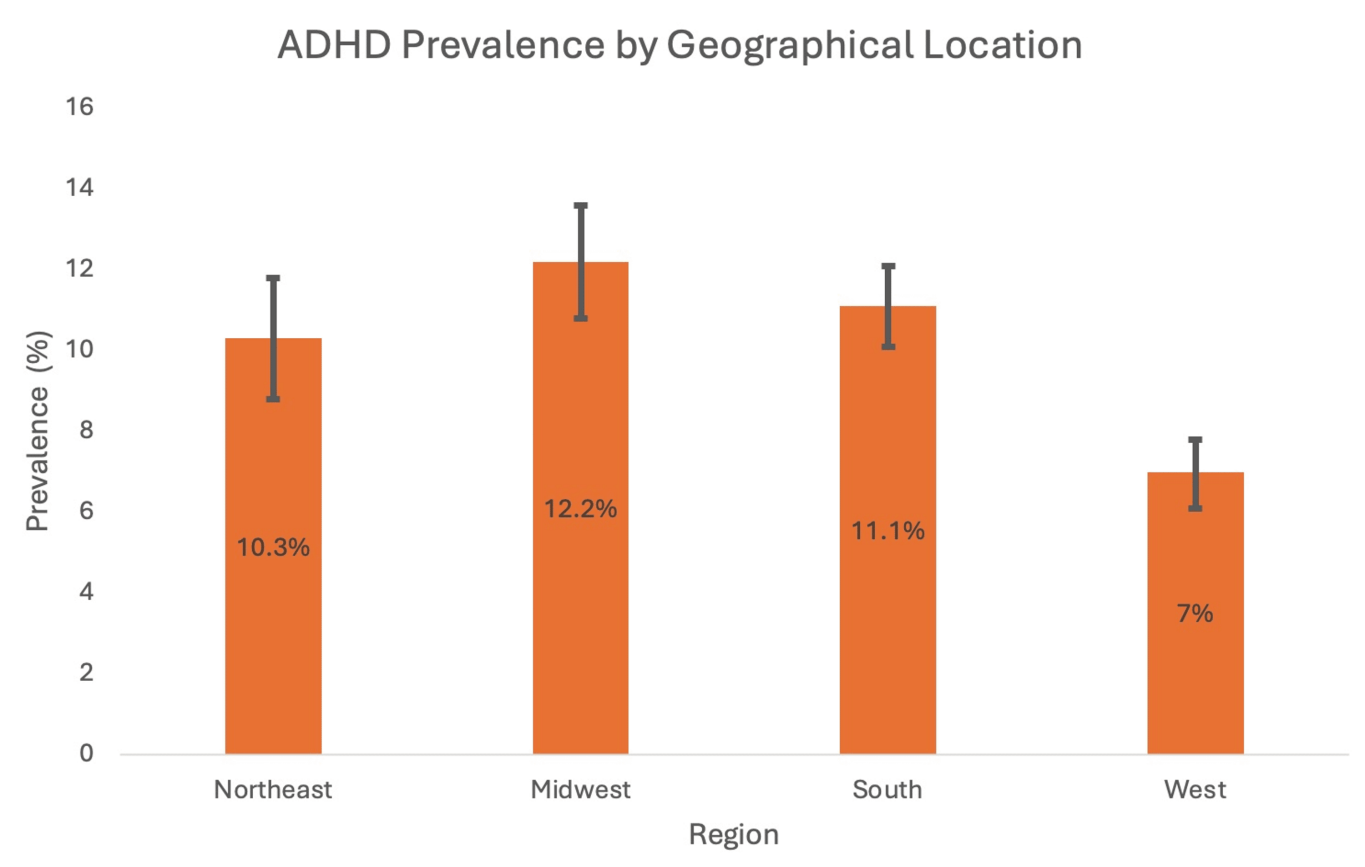
ADHD prevalence in Northeast, Midwest, South and West United States with 95% CI intervals (2015-2016) ADHD: attention deficit hyperactivity disorder Source: [12]

A cross-sectional study conducted by Xu et al. (2018) analysed data from the United States national interview health survey from 1997 to 2016 to calculate temporal trends in ADHD prevalence amongst individuals aged 4 to 17 [12]. The data showed a continuous increase in ADHD prevalence from 1997 to 2016. Out of the 186,457 participants, a total of 14,704 reported receiving a formal diagnosis of ADHD during this period. The study found that in the year 2015-2016, the prevalence was 10.2% (P<0.001) compared to 6.1% in the year 1997-1998 (P<0.001). The researchers of the study organised the data by race, geographical location, socioeconomic status (SES) and age in order to conduct a subgroup analysis, which found that the increase in ADHD prevalence was statistically significant in all of these subgroups [12]. Figures 1-4 show ADHD prevalence across the different subgroups.

Variational Patterns in ADHD Prevalence

This study, conducted by McKechnie et al. (2023), aimed to calculate the prevalence of ADHD diagnoses and ADHD prescriptions in the UK from 2000-2018 among different subgroups of people based on variables such as SES and age [21]. From 2000 to 2018, the study had 7,655,931 participants with at least one full calendar year of data, of whom 35,877 (0.5%) had a formal diagnosis of ADHD and 18,518 (0.2%) had received prescription ADHD medication. The study found that the rates of ADHD diagnoses and ADHD prescriptions have been increasing proportionally among all ages from 2000 to 2018, except for 3-5-year-olds, in whom diagnoses declined by about 50% in boys and 33% in girls.

Furthermore, the study also found that children, particularly aged 6-9 years, experienced the greatest absolute increase (20-fold) in ADHD prevalence, whereas adults, especially men aged 18-29, experienced the greatest relative increase (50-fold) during the period of the study. This study also found that the ratio of males to females with a new diagnosis of ADHD varied across different ages. In the 13-15 years age group, the ratio of men to women with a new ADHD diagnosis was 4:1; however, this was inversely related to increasing age as the ratio decreased to 2:1 for 16-29 years, 1.5:1 for 30-39 years and 1:1 for 40 years or older [21]. There was a higher prevalence of ADHD diagnoses and prescriptions amongst certain groups of people, such as men compared to women and children compared to adults. These findings are in keeping with the results of other studies on ADHD prevalence. Interestingly, the study also found a statistically significant association between SES and ADHD prevalence, as the most deprived areas had almost double the rates of ADHD diagnoses and prescriptions compared to the top 20% of least deprived areas [21]. The possible reasons to explain this will be, lower SES may be linked to higher exposure to risk factors during the pre- and peri-natal period which may have a causal effect on the ADHD development (such risk factors include toxins, maternal stress and poor nutrition) and parents with ADHD are more likely to be socioeconomically disadvantaged due to their ADHD traits, therefore children may inherit these ADHD traits and be disadvantaged themselves. Table 1 shows the prevalence of ADHD in 2018 among different subgroups of age and sex [21].

**Table 1:** Prevalence rates of ADHD diagnoses and ADHD prescriptions in men, women, boys, and girls in 2018 Source: [21]

Group	ADHD prevalence (per 10,000)	95% CI (%)	Prescriptions (per 10,000)	95% CI (%)
Boys	255	247-263	156	150-163
Girls	67.7	63.5-71.9	36.8	33.8-40.0
Men	74.3	72.3-76.2	13.3	12.5-14.1
Women	20	19.0-21	4.5	4.1-5.0

Variation of ADHD Prevalence Over a 10-Year Follow-Up Period

Chung et al. (2019) analysed data from the Kaiser Permanente Northern California health plan to calculate the time trends in ADHD prevalence over 10 years amongst different racial/ethnic groups [22].

In this study, a total of 6,150,330 (5,282,877 adults and 867,453 children) participant records were studied over the years 2007-2016. The prevalence of ADHD in adults increased from 0.43% in 2007 to 0.96% in 2016, whereas for children, aged 5 to 11 years, it increased from 2.96% (2007) to 3.74% (2016). Throughout the study, the prevalence of diagnosed ADHD was 1.12% in adults versus 4.78% in children. The study also found that the rate of new annual adult ADHD diagnoses per 10,000 persons increased significantly from 9.43 in 2007 to 13.49 in 2016 [22].

This study conducted a subgroup analysis and found variation in the prevalence trends amongst adults by ethnicity/race group, although prevalence increased in all groups. The greatest increase in prevalence was amongst White individuals, which increased from 0.67% (2007) to 1.42% (2016); contrastingly, the increase in ADHD prevalence was lower for Black or African American individuals (0.22% in 2007 to 0.69% in 2016) and Hispanic individuals (0.25% in 2007 to 0.65% in 2016) [22]. There were also associations between certain demographics and the odds of having an ADHD diagnosis. For example, the odds of having an ADHD diagnosis in women compared to men were 0.943 (95% CI, 0.928-0.959), and the odds of Asian patients compared to White patients were 0.248 (95% CI, 0.240-0.257). Other factors which were found to increase the odds of ADHD diagnosis were divorced marital status, unemployment, and younger age. In addition, this study found strong associations between ADHD and other psychiatric conditions. For example, the odds of having an ADHD diagnosis with an eating disorder, bipolar disorder and anxiety disorder were 15.9, 8.6 and 6.0, respectively [22].

Global Prevalence of ADHD in Children and Adolescents

This study was a systematic review carried out by Ayano et al. (2023), which consisted of 3,277,590 participants and aimed to estimate the global prevalence of ADHD in children and adolescents [23]. Across all the studies examined in this review, the prevalence of ADHD was found to be 8.0% (95% CI: 6.0-10%). This systematic review includes data from children and adolescents with different characteristics, such as demographics, geography, etc. Therefore, a subgroup analysis was conducted on the pooled data. Study found that ADHD prevalence in boys was 10% (95% CI: 8-11%) was statistically significantly double the prevalence in girls, 5% (95% CI: 4-7%).

The study also found variations in prevalence by ADHD subtypes. ADHD-hyperactive had a prevalence of 2.95% (95% CI: 1.8-4%), ADHD-inattentive had a prevalence of 3% (CI 95%: 2.0-4%), and ADHD-combined had a prevalence of 2.44% (95% CI: 1.5-3.5%). Although this data shows the inattentive subtype is most prevalent, followed by the hyperactive and combined subtypes, the variational patterns seen here are not statistically significant, as there is significant overlap between the confidence intervals [23]. This systematic review also found patterns in ADHD prevalence when the data were subgrouped by age. The prevalence in children between the ages of 6-11 was found to be 7%, which is over double compared to 3% in adolescents aged 12-18 years. Furthermore, the study did not find statistically significant variation in ADHD prevalence amongst children and adolescents across different countries, with the reported prevalence rate ranging between 6 to 7.5% [23].

This study also found the prevalence of ADHD to vary depending on whether the data were taken from a clinical (e.g. hospitals) vs. nonclinical settings (e.g., schools), although this was not statistically significant due to overlap between confidence intervals. Prevalence of ADHD based on data from a clinical setting was 8.74% (95% CI: 5.66-13.27%), whereas prevalence based on community setting derived data was 7.19% (95% CI: 5.59-9.19%). The study also found that reported ADHD prevalence amongst studies had a positive correlation to study size, as larger studies report higher prevalence figures [23].

Does the Prevalence of the ADHD Phenotype Correlate With the Prevalence of ADHD Diagnoses?

A study conducted by Rydell et al. (2018) recruited 19,271 9-year-old participants and followed them up over 10 years from 2004-2014 [24]. Parents were interviewed using a pre-made questionnaire to identify whether participants had ADHD traits based on DSM-IV criteria. Their answers to the questions were used to score participants on an ADHD scale, which had cut-off scores for both diagnostic-level ADHD as well as subthreshold ADHD [24].

The study found that the prevalence of diagnostic level ADHD amongst the participants remained around 2% over the 10-year study period with no statistically significant changes (OR 1.37; 95% CI: 0.77-2.45; p-value 0.233), whereas the prevalence of both subthreshold ADHD and average ADHD scores significantly increased over the study period (p<.001). However, over the 10 years, the prevalence of clinically diagnosed ADHD within the population increased five-fold (OR 5.27, 95% CI: 1.85-14.96) despite no increase in diagnostic level ADHD based on questionnaires used in the study. This suggests that the increase in clinically diagnosed ADHD is influenced by various external factors rather than a true rise in the ADHD phenotype [24].

The researchers of the study Jin et al. (2014) aimed to identify factors contributing to variation in ADHD prevalence amongst 5-15-year-old children in Shanghai, China [25]. This study utilised questionnaires to identify participants who are more likely to have ADHD, followed by interviews with these higher-risk individuals and their parents, using DSM-IV-derived criteria to determine which participants had diagnostic-level ADHD [25]. Of the 9,900 valid questionnaires utilised, the prevalence of ADHD was 4.6% of which 2.4% had ADHD-inattentive, 0.4% had ADHD-hyperactive, and 1.8% had ADHD-combined subtype. This shows the prevalence rate varies with ADHD subtype, with ADHD-inattentive being most common in this study [25].

Furthermore, prevalence was found to vary with gender as boys had a higher prevalence (6.6%) compared to girls (2.7%), which is in keeping with the gender disparities in ADHD prevalence found by multiple studies across the literature. The study also highlights that age group affects ADHD prevalence as participants aged 7-10 years exhibited the highest prevalence (6.3%) compared to children from any other age group [25]. In addition, the study revealed that geographical location has an influence on ADHD prevalence, as children from rural areas were noted to have a higher prevalence compared to children living in city areas. Foreign families who immigrate to city areas may better integrate into mainstream society, which may reduce reported ADHD prevalence. Interestingly, the study also found socioemotional status to be a significant factor causing variations in ADHD prevalence, as children from low-income households were found to have statistically significantly higher ADHD prevalence [25]. Foreign resident parents may have a higher level of income and education, which may correlate to higher expectations from children and more protective parenting practices.

All of this could reduce reported ADHD prevalence in the city relative to outside of the city [25].

Factors Driving ADHD Prevalence

Skounti et al. (2007) and colleagues found that there is significant variation in ADHD prevalence in the literature, with rates ranging from as low as 2.2% in some studies to as high as 17.8% in other studies [26]. It aimed to explain this variation [26].

The literature review identified variations in ADHD prevalence between boys and girls. Boys were reported to have higher prevalence rates compared to girls in both community and clinical settings, with the difference being more pronounced in clinical settings; in the clinical setting, the male-to-female ratio was as high as 9.1 compared to the ratio ranging between 1:1 and 1:3 in the community-based setting. The review also found that prevalence was influenced by age, with ADHD rates declining as individuals get older. For example, ADHD prevalence was significantly lower in the 17-20 age group (6.0%) compared to the 10-13 age group (12.8%) [26].

It was also identified that geographical location had an influence on ADHD, as individuals residing outside of the city areas had higher reported rates of ADHD. Likewise, SES also had effects on ADHD rates, with individuals from lower-income backgrounds having significantly higher prevalence rates of ADHD. 

This review found that data on the effect of ethnicity on ADHD prevalence is both mixed and limited, with some studies reporting lower ADHD rates in Black or African American children compared to White children, whilst other studies report lower rates among certain ethnicities such as Hispanic and Asian children [26]. Prevalence rates also varied depending on the source of information used to assess children for ADHD. Studies that relied on teacher assessments and school-based reports showed a higher prevalence of ADHD compared to studies that used parent-based interviews or symptom questionnaires answered by the children themselves. Furthermore, studies that utilised more than one source of information reported lower prevalence rates (2.2% to 16.1%) compared to studies that only used a single source (2.8% to 17.8%). This shows that the methodological procedures used in the study can influence the reported prevalence [26].

The diagnostic criterion used to assess children for ADHD also affected the prevalence rate, as studies that used DSM-IV criteria reported higher rates compared to studies that used DSM-III-R; this is due to evidence-based changes in the diagnostic criteria, which lead to higher sensitivity for identifying ADHD. Furthermore, when study criteria included assessment of impairments, the prevalence rate was significantly lower (6.8%) compared to when impairment was considered in assessing participants for ADHD (16.1%) [26].

Variation was noted in ADHD prevalence by subtype, with ADHD-I (inattentive) being the most prevalent, followed by ADHD-C (combined) and ADHD-HI (hyperactive-impulsive). Interestingly, variations in gender were also found across the subtypes; even though boys had a higher prevalence rate in all subtypes, the percentage of girls exhibiting symptoms in the ADHD-I subgroup was significantly higher than in ADHD-C and ADHD-HI [26].

Combined Effect of Socioeconomic Status and Parental History 

The study conducted by Rowland et al. (2018) aimed to understand how SES and parental history of ADHD interact to influence ADHD prevalence [27]. The study found that the odds of children from low-income backgrounds having ADHD were 6.2 times greater (95% CI 3.4-11.3) compared to those of children from high-income backgrounds, which is in keeping with numerous other studies across the literature.

The study also found that the odds of ADHD were 10 times greater in all children with a parental history of ADHD compared to children without a parental history of ADHD, irrespective of their SES. Interestingly, among the children with a parental history of ADHD, SES was found to have a significantly smaller effect on ADHD status. In a background of parental ADHD history, the odds of ADHD in children from a low socioeconomic background were only 1.4 times greater compared to children from a high socioeconomic background. This result directly shows that in a background where both factors exist, the parental ADHD status becomes a bigger predictor of ADHD prevalence compared to socioemotional status [27]. The SES gradient is less pronounced when there is a parental history of ADHD. The authors state this may be because environmental risk factors (such as SES) are far less influential when there is a background of genetic susceptibility. In contrast, when there is an absence of such genetic vulnerability, the incremental effect of SES is significantly stronger. Furthermore, parents who have an ADHD diagnosis may have increased awareness and access to diagnostic services [27].

Geographical Variation in ADHD Prevalence

A Danish study: The study led by Madsen et al. (2015) analysed data from several different Danish municipalities to determine inter-municipality geographical variation in ADHD prevalence and examine potential responsible factors [28].

The study found significant geographical variation across the municipalities; some areas had prevalence rates as little as 0% whereas other areas had rates of up to 2.7%. The study revealed that areas with lower population densities (which tend to have limited access to diagnostic resources) have higher rates of ADHD in comparison to high population density areas (which often have greater access to diagnostic resources), despite the Danish healthcare system being a free, tax-funded system. Despite the study showing a correlation between access to healthcare services and ADHD prevalence, when the data were analysed using Bayesian spatial regression models, no statistically significant associations were found between ADHD prevalence and (1) municipality spending on children’s healthcare, (2) socioeconomic status, (3) access to diagnostic services or (4) different diagnostic practices. Therefore, the study concludes that even though the correlation between geographical location and ADHD prevalence is not statistically associated with any of the four contextual factors, it likely still plays a small role in a complex background of multiple factors interacting with each other to determine ADHD prevalence. This is in contrast to the geographical variation of ADHD prevalence seen across the USA, which has statistically significant associations with contextual factors such as spending on healthcare and SES [28].

A Norwegian study: Widding-Havneraas et al. (2023) conducted a Norwegian study which aimed to determine if the geographical variation in ADHD diagnoses corresponds to variation in ADHD symptoms [29]. This would explain whether ADHD geographical variation is a true reflection of ADHD symptoms or whether it is influenced by alternative factors [29].

Firstly, the researchers obtained registry data from 63 clinics in Norway to determine the prevalence of formally diagnosed ADHD at the clinic level. Secondly, the researchers obtained data on ADHD symptoms in the catchment areas of those clinics using a nationwide survey. The compiled data were analysed for cross-sectional associations between prevalence of ADHD diagnoses and prevalence of ADHD symptoms, which revealed that geographical variation in ADHD diagnoses is significantly greater than the variation in ADHD symptoms [29].

The study concludes that geographical variation in ADHD diagnoses is far greater than can be explained by variation in ADHD symptoms and is therefore likely influenced by other external factors (for example, disparities in diagnostic practices). The study notes that Norway has a state-funded healthcare system, which is free at the point of access, hence it is unlikely that access to diagnostic services is a significant factor in geographical variation of ADHD [29].

Estimates of ADHD Prevalence Across Three Decades

It has been thought for a long time that variations in ADHD prevalence reported by different studies are due to methodological disparities in how the research was conducted (e.g., criteria used to diagnose ADHD). However, in recent times, the rate of clinically diagnosed ADHD has been increasing, which raises the question of whether this is due to an increase in the prevalence of the ADHD phenotype or an external factor [13].

The systematic review led by Polanczyk et al. (2014) found that the variations in ADHD prevalence amongst different studies were influenced by various factors [13]. The study model accounted for several of these variables, including diagnostic criteria and the source of information used to identify ADHD. The final model showed that 44.4% of the variation in prevalence figures could be explained by the factors used in the study, particularly methodological differences amongst studies (P<0.05) [13].

ADHD prevalence was 5.47% (P=0.003) higher when the study relied on teacher assessment to identify participants with ADHD compared to when the prevalence figures were based on comprehensive assessments. Studies that required evidence of impairment to classify participants as ADHD reported prevalence rates that were 2.3% lower (P=0.018) than studies which didn’t require impairment. Review found that the diagnostic criteria played a role in the variation in prevalence rates. Studies that used the DSM-IV criteria instead of DSM-III-R or ICD-10 reported prevalence rates of 2.42% (P= 0.044) and 4.09% (P=0.009) higher, respectively [13].

The univariate analysis found a number of variables which had statistically significant effects on ADHD prevalence, including the method used to assess for ADHD symptoms, the diagnostic criterion used, etc. However, the year in which the study was published did not have a significant effect on ADHD prevalence (P=0.68). Furthermore, multivariate analysis also found that many of the previously discussed variables account for heterogeneity in reported prevalence rates, except for the year of publication, which was still found to be insignificant in accounting for the variations in ADHD prevalence over time. This study concludes that when differences in study methods are controlled for, the reported prevalence rate does not vary over the past three decades. The review also found that geographical variation did not play a significant role in the variation of prevalence rates (P value between 0.172 and 0.651) when study differences were accounted for [13].

A Systematic Review of ADHD Prevalence Amongst Children and Adolescents in China

Wang et al. (2017) led a systematic review exploring ADHD prevalence among children and adolescents in China [30]. The study found that the pooled prevalence across 67 studies with a total of 275,502 individuals was 6.26% (95% CI: 5.36-7.22%) [30]. Significant variation in ADHD prevalence was noted across ADHD subtypes, genders and diagnostic criteria used. This data is tabulated in Tables 2-4. The study found that ADHD prevalence is significantly higher in men than women (P=0.001%). The study also found statistically significant differences in ADHD prevalence between DSM-III and DSM-IV (P<0.05) as well as between DSM-III and DSM-5 (P<0.05). Significant heterogeneity was noted across studies (I2 = 99.0%, P < 0.001). Subgroup analysis revealed that geographical location and "source of information" explained some of the heterogeneity [30].

**Table 2:** ADHD prevalence organised by ADHD subtypes This table shows that ADHD-I (inattentive) is the most common, followed by ADHD-C (combined) and ADHD-HI (hyperactive-impulsive), although there is overlap of confidence intervals. ADHD: attention deficit hyperactivity disorder Source: [30]

ADHD subtype	Prevalence (%)	95% CI
ADHD- I	3.24	2.52-4.04
ADHD-HI	1.16	0.87-1.48
ADHD-C	1.71	1.33-2.13

**Table 3:** ADHD prevalence organised by gender This table shows that ADHD prevalence is higher in males. ADHD: attention deficit hyperactivity disorder Source: [30]

Gender	Prevalence (%)	95% CI
Male	8.17	6.94-9.50
Female	6.22	5.07-7.48

**Table 4:** ADHD prevalence organised by diagnostic criteria utilised This table shows that there is significant variation in ADHD prevalence when different criteria are used. ADHD: attention deficit hyperactivity disorder; DSM-III, DSM-III-R, DSM-IV, and DSM-5: Diagnostic and Statistical Manual of Mental Disorders-Third Edition, Third Edition-Revised, Fourth Edition, and Fifth Edition. Source: [30]

Diagnostic criteria	Prevalence (%)	95% CI
DSM-III	4.27	3.50-5.11
DSM-III-R	6.85	4.21-10.06
DSM-IV	6.36	5.17-7.67
DSM-5	5.91	5.09-6.79

Epidemiological Nationwide German Study 

Akmatov et al. (2018) and colleagues analysed a nationwide dataset containing 6 million children from Germany to calculate trends and variation in ADHD prevalence from 2009-2016 [31]. In 2016, 260,000 of the 6 million children were diagnosed with ADHD, resulting in a 4.33% prevalence rate [31].

Geographical disparities in ADHD prevalence were noted as prevalence rates were lower in Northern and Southern Germany. Although the geographical variation in ADHD prevalence became less pronounced over the duration of the study, it is still statistically significant. Inter-district ADHD variation was associated with access to diagnostic services, as areas with a higher number of physiatrists reported a higher prevalence of ADHD. Furthermore, children who migrated to a district with a higher ADHD prevalence were 1.36 times more likely to be diagnosed with ADHD, which suggests that access to diagnostic care could be a determinant of ADHD prevalence [31].

From 2009-2016, the study did not find any statistically significant increase in ADHD prevalence, which is in keeping with the findings of numerous similar studies. There were also gender and age disparities in ADHD prevalence. The likelihood of ADHD diagnosis was three times greater in boys compared to girls. ADHD was also more likely to be diagnosed in older children until the age of 11, after which the incidence of ADHD became increasingly unlikely [31].

Discussion

ADHD Diagnostic Criteria Changes 

There is substantial evidence to suggest that changes in diagnostic criteria are to play a significant role in the increasing ADHD prevalence rates [10,32]. The first time an ADHD-like syndrome was introduced in the DSM was in its second edition in 1968, whereby it was called and described as "hyperkinetic reaction of childhood" in a single sentence. Although this syndrome of hyperkinetic reaction of childhood encompasses many of the features of ADHD as we know it today, it places a larger emphasis on symptoms of hyperactivity whilst neglecting symptoms of inattentiveness, which leads to a significant proportion of people with predominantly inattentive symptoms being excluded from being from the diagnosis [33].

In the third edition of the DSM, the syndrome was renamed to attention deficit disorder (ADD) and included two subtypes consisting of ADD with and without hyperactivity, giving recognition to people without hyperactive symptoms. Furthermore, the DSM-III was the first to define characteristics of the condition, such as age of onset, symptom duration and specific criteria for diagnosis. As the DSM was revised over time, there was a shift to improve recognition of the inattentive features of ADHD, which were previously neglected [34].

Therefore, we see that previous editions of the DSM overemphasise or underemphasise the importance of the different types of ADHD symptoms, thereby excluding groups of individuals from being recognised as having ADHD, whilst some editions of the DSM, such as DSM-I, very poorly define ADHD, which can lead to poor recognition of the syndrome.

Then, in the DSM-III-R, the syndrome was again named to the familiar term of ADHD, and the distinction between subtypes was removed and instead replaced with a single criterion which encompassed symptoms of hyperactivity, impulsivity, and inattentiveness. Finally, in the DSM-IV (the latest edition of the DSM), ADHD was split into three subtypes: ADHD-I, ADHD-HI and ADHD-C. By categorising ADHD by the different domains of its symptoms, it ensures each domain of symptoms is given significant emphasis and allows for improved recognition and diagnosis by better defining the broad symptomatology of the condition [35].

Table 5 shows major changes in the DSM criteria between the fourth and fifth editions. These changes reflect a broader and inclusive criterion for ADHD in the fourth edition, which may explain the increase in ADHD diagnosis rates [35].

**Table 5:** Changes from DSM-IV to DSM-5 The table shows that autism is no longer an exclusionary diagnosis, which is significant as autism spectrum disorder (ASD) and ADHD are heavily comorbid. Therefore, the DSM-IV excluded a significant number of patients being diagnosed with ADHD. ADHD: attention deficit hyperactivity disorder; DSM-IV and DSM-5: Diagnostic and Statistical Manual of Mental Disorders, Fourth and Fifth Editions Source: [35]

DSM criterion	DSM-IV	DSM-5
(A) Number of symptoms required	6 or more	6 or more if under 17 years 5 or more if over 17 years
(B) Age of symptom onset	Must have symptom onset before 7 years	Must have symptom onset before 12 years
(C) Pervasiveness	Evidence of impairment	Evidence of symptoms in 2 or more settings
(D) Impairment	Must be "clinically significant"	"Reduce the quality of social, academic or occupational functioning"
(E) Exclusionary Conditions	Includes ASD as an exclusionary diagnosis	Does not include ASD as an exclusionary diagnosis

Gender Disparities in ADHD Prevalence 

ADHD prevalence has been found to be significantly more prevalent in boys compared to girls consistently throughout the literature [36,37]. Although these disparities have existed for a long time, recently ADHD prevalence rates have been increasing at a faster rate in girls, though prevalence is still more common in boys overall [16]. This trend is partly due to increased awareness of ADHD amongst underrepresented groups, such as girls, due to ADHD becoming increasingly popular in the media. Furthermore, healthcare professionals are also becoming increasingly well-versed with ADHD as our scientific understanding of the syndrome grows [12].

Another major contributor to this temporal trend of increasing prevalence in girls is due to changes in the DSM criteria which placed a focus on the attentive deficits seen in ADHD which were neglected in previous DSM editions; girls are more likely to display inattentive symptoms rather than hyperactive symptoms therefore changes in DSM criteria mean girls are more likely to be recognised and diagnosed by healthcare professionals [15,38]. The reason for this difference in symptomatology between boys and girls is thought to be due to social factors, for example, girls are more likely to be forced to sit quietly in class due to social pressures compared to boys, who are more likely to exhibit symptoms of hyperactivity; this leads to missed diagnoses in girls and early recognition and treatment in boys [39]. However, other studies have found that boys and girls exhibit similar levels of hyperactive symptoms, but the symptoms in boys are given more weight than in girls due to social biases in individuals [40]. For example, teachers were less likely to respond to hyperactive symptoms in girls compared to boys [41,42]. As healthcare professionals become increasingly aware of these gender disparities and differences in symptomatic presentation between boys and girls, they are more likely to be aware of and consider their biases and ask more specific questions to identify key features of ADHD in girls, such as predominantly inattentive symptoms [43].

Race and Ethnic Disparities in ADHD Prevalence 

Studies consistently show that the prevalence of ADHD diagnoses is lower in persons of colour (POC), especially Black people, compared to White people, even after accounting for confounding variables such as SES [44]. One of the reasons for this is that ADHD is largely a subjective diagnosis, which is mainly made through interviews rather than quantitative biomarkers. This method of diagnosis relies on physicians asking the correct type of questions and being receptive to patients' answers from an unbiased perspective. Although there are various diagnostic criteria and aids available to assist physicians in diagnosis-making, these aren’t universally used, and there is variation within these diagnostic tools themselves; in essence, there is no standardised method to diagnose ADHD. 

Due to the subjectivity in ADHD diagnoses, a lot of weight is placed on how physicians respond to and interpret information they receive from patients themselves, teacher-based reports, parent interviews, etc.; therefore, it is possible that their personal biases can influence diagnosis [45]. For example, studies found that physicians are likely to be more receptive to White parents who come to the office to discuss their child’s ADHD symptoms [46]. Furthermore, such biases are not specific to ADHD but rather are seen across many mental health conditions, suggesting that personalised biases within healthcare professionals may be a broader issue beyond the scope of ADHD [47].

Despite the racial disparities in ADHD prevalence, the rate at which Black or African American students are being diagnosed with ADHD in the recent decade is three times greater compared to White students [48]. Likewise, there is an increasing rate of diagnoses in other under-represented groups, such as girls and other ethnic minorities, which is thought to be a major driver in the overall increasing prevalence of ADHD diagnoses [48,49].

This is largely due to increasing awareness of ADHD amongst people due to its increased coverage in the media, which enables people from these under-represented groups to gain more insight into their symptoms and ultimately seek medical attention, therefore leading to them being diagnosed [14]. Whilst social media is positive in the sense that it allows individuals to gain access to accurate information about ADHD from licensed healthcare professionals, another study found that approximately 50% of information on social media regarding ADHD is inaccurate [50]. Therefore, whilst social media has a major role in increasing public awareness on ADHD, there is also potential harm being caused by misleading information, which serves as an area for future areas of improvement.

Furthermore, since 2004, the month of October has been designated as ADHD awareness month, which is internationally recognised. During October, there is a focus on educating the public on ADHD and dismantling harmful stereotypes and misinformation. In addition, Google searches on ADHD have increased significantly in the recent decade, showing that the public is becoming ever more aware of this clinical syndrome. In addition to the public, healthcare professionals are also becoming increasingly well-versed with ADHD, partly due to research uncovering ADHD pathology and increasing awareness of disparities and biases in ADHD diagnoses [12].

In summary, increasing public awareness is causing more people to seek attention for their symptoms, leading to individuals who otherwise wouldn’t have been previously diagnosed finally gaining a formal diagnosis. Therefore, it is likely that although ADHD diagnosis rates are increasing due to this phenomenon, the actual prevalence of the ADHD phenotype is relatively constant. Healthcare professionals should assess each patient holistically and be aware of common biases to avoid them and ensure thoughtful diagnosis.

Geographical and Socioeconomic Disparities 

The majority of studies found that there is no statistically significant association between geographical location and ADHD prevalence, as the differences are largely attributed to differences in diagnostic methods. For example, a large meta-analysis found that the slightly higher prevalence in North America (6.8%) compared to Europe (4.6%) was due to the fact that the former predominantly used DSM criteria, whereas the latter used ICD-10 criteria [51]. The ICD-10 criteria are significantly stricter than DSM-IV as they require symptoms in all three domains of ADHD symptoms compared to one domain for DSM-IV. ICD-10 also has a list of conditions which cannot co-occur with ADHD, hence excluding a child with such a condition from meeting the diagnostic criteria, whereas DSM-IV allows for these conditions to co-exist with ADHD [52].

Furthermore, it is likely that disparities in diagnostic methods also account for variation in ADHD prevalence internationally; a large multi-country study, which aimed to obtain prevalence values across clinics in Africa, Europe, North America, and Australia using a uniform diagnostic tool, found no statistically significant variation in prevalence [53]. This data leads to the question of whether ICD is a more restrictive criterion and therefore is more likely to underdiagnose, whereas DSM is relatively more lenient and therefore more likely to overdiagnose, and the answer is not known. This information highlights the need for a more unified approach to diagnosing ADHD, as overdiagnosis or underdiagnosis can have significant functional impacts on affected individuals. Effects of inaccurate diagnoses stretch beyond social functioning, as the annual ADHD cost for individuals is estimated to be $1000 more compared to age-matched subjects, which presents a financial burden on those affected [54].

Low SES has been associated with increased ADHD prevalence in many studies throughout the literature. This association is likely mediated by confounding factors associated with low SES, for example, smoking during pregnancy, which is known to be an environmental risk factor for ADHD [55]. ADHD requires physician access for diagnosis; hence, low SES individuals are more likely to face barriers in accessing diagnostic services and care due to high costs [27]. Russel et al. (2015) hypothesised that low parental SES is a result and reflection of parental ADHD traits, which may have led to poor academic attainment and/or unstable employability [56]. Therefore, the children of low SES parents are more likely to have ADHD due to inheritance of ADHD-associated genes rather than the SES parents [56]. Kvist et al. (2013) concluded that having a child with ADHD reduces parental SES by dissolving their relationship and reducing their labour supply, rather than low SES being a risk factor for ADHD itself [57]. The study found parents of ADHD children were 75% more likely to have their relationship terminated and 7-13% more likely to have diminished labour supply following 10 years from the birth of the first ADHD-diagnosed child [57]. However, other studies do not support this reverse causality relationship [58].

Consequences of ADHD Undertreatment

As ADHD diagnosis rates grow, there is increasingly more discussion about overdiagnosis despite evidence that the actual prevalence of the ADHD phenotype remains constant. Labelling ADHD as an over-diagnosed condition is harmful as it can increase stigma and pose additional barriers to those wanting to seek medical attention for ADHD-like symptoms by de-medicalising ADHD. This can lead to undertreatment, which can pose serious consequences to such individuals [59].

ADHD can have debilitating effects throughout the lives of those affected by the condition. Starting from primary school age, ADHD can have significant effects on academic achievements in children with the syndrome [60]. Furthermore, the effects on ADHD in children extend to their families, with parents reporting stress levels far greater compared to parents of children without ADHD [61]. In 50-60% of ADHD children, symptoms carry over into adolescence, with up to 20% of them reporting severely dysfunctional impairment in their routine lives. As a result of the increase in perceived stress in adolescents with ADHD, there is a significantly increased risk of substance abuse in these individuals, which presents with its own set of legal, societal and health challenges to these individuals [62]. The stressful effects are even more pronounced in underrepresented groups, such as girls, as they are less likely to be diagnosed and treated as early as boys and also experience increased perceived stress compared to boys [11]. Furthermore, up to 60% of ADHD individuals will have symptoms which persist in adulthood, which manifest in various settings such as employment and relationship stability; the risk of employment redundancy is significantly increased relative to national rates of dismissal [63,64]. In addition, due to the strong genetic component of ADHD, affected individuals have a far higher risk of the disorder being present in their children, which poses further long-term familial challenges [65].

Challenges in ADHD Treatment as Prevalence Grows 

As prevalence rates of ADHD diagnoses increase, so does the use of ADHD medications, which pose their own challenges. ADHD medications fall into two main categories, stimulants and non-stimulants. The primary treatment for ADHD is stimulant medications, which include amphetamine and methylphenidate formulations; these medications have some very common side effects, such as lack of appetite, insomnia, and nausea [64] [66]. Furthermore, there are concerns regarding the three main long-term effects of stimulants. 

Firstly, stimulants are known to cause long-term suppressive effects on growth and development in up to 20% of children receiving active treatment. Although initial treatment causes a reduction in weight, long-term use causes a significant increase in BMI, which poses additional health risks associated with being overweight, thereby adding to the health burden already placed on ADHD individuals [67,68]. Some physicians aim to minimise this effect for their patients by implementing a "structured treatment interruption", also known as a drug holiday. This is when a child stops taking their medication for a period of time under the supervision of their doctor. However, evidence shows that the adverse functional effects of these drug holidays often outweigh their benefits [69,70]. Secondly, there is some evidence which shows that ADHD medication increases substance abuse risk; however, this is controversial due to mixed evidence. Some people think that the euphoric effects of ADHD medications can cause patients to become dependent, which may lead them to seek similar effects in other drugs; however, studies show that this is not the case and that ADHD medication may actually decrease substance abuse risk [71].

Conversely, studies do show that stimulants come with significant diversion potential, where individuals can misuse their medications, for example, to increase academic performance [72]. Data show that ADHD stimulant misuse and diversion are a common healthcare issue [71]. Studies show that the lifetime risk of stimulant diversion amongst ADHD individuals is as high as 24-26% of patients selling, trading or sharing their medications, although there is variation between studies [73-75]. Lastly, given the sympathomimetic effects of stimulants, some small-scale prospective studies show a link between stimulant use and adverse cardiac outcomes; however, more recent large-scale studies do not support this link [76,77]. Therefore, as ADHD prevalence grows, the adverse effects of these medications will become more apparent on a larger scale and pose public health concerns. Hence, future improvement of ADHD medications in terms of side effect profile is a key area of development [32]. Whilst randomised control trial (RCT) data show compelling evidence for short-term ADHD medication efficacy, there is a lack of evidence to support the long-term efficacy of current primary ADHD medications. Long-term prospective cohort studies show that the naturalistic effects of treatment are not statistically significant, especially when considering functional outcomes such as social function and academic achievement [78]. For example, a meta-analysis consisting of 34 trials on the efficacy of methylphenidate found that the medication was not consistent, nor was it significant in improving academic outcomes [79].

Contrastingly, RCT data are compelling for improvement in short-term outcomes. For example, data from a Swedish national registry revealed a decrease in crime rate shortly after an increase in stimulant use. Similarly, data from American national registries show an inverse relationship between stimulant use and vehicle accidents [80,81].

This discrepancy in short-term RCT efficacy and long-term naturalistic effects of ADHD medication can be partly explained by two factors. Firstly, prolonged use of stimulants can cause patients to develop tolerance to them, which is a major flaw, as people with ADHD usually need to take these medications for up to a lifetime [82]. Secondly, naturalistic follow-up studies show that long-term adherence to stimulants is relatively low, perhaps due to their side effects. whereas in clinical trials, adherence is very high [83].

Therefore, as ADHD prevalence grows, the treatment demands will also grow, which will pose challenges as current treatments do not show compelling naturalistic long-term efficacy and are also accompanied by a variety of common side effects. Therefore, in the interest of public health, continued research is needed to formulate better pharmacological treatments for ADHD.

Strengths and Limitations

Variability in diagnostic criteria: There is significant variability in the diagnostic criteria among different studies. The two main criteria used are DSM-IV and ICD-10, and there are significant differences between the two. Ultimately, DSM criteria are more lenient than ICD-10, which accounts for a significant amount of variability in reported prevalence figures. More specifically, studies utilising DSM are more likely to report increased prevalence compared to studies utilising ICD-10 [2]. This heterogeneity reduces comparability across studies. In addition, some studies use different editions of the criteria’s such as DSM-IV and DSM-5, whilst other studies use their own scoring systems to determine ADHD status, which may not accurately reflect diagnostic criteria. 

Sources of Data

Parent interviews and teacher-based reports: Some of the studies rely on parent interviews to determine the ADHD status of children by asking parents whether children were ever diagnosed to have ADHD by a healthcare professional. This introduces recall bias as parents may not be able to accurately recall this information, which leads to over- or underreporting of ADHD diagnoses, thereby hindering the reliability of the results [24,25]. Furthermore, studies which rely on subjective sources of information (e.g. teacher reports) without following up with objective measures, such as clinical assessments and health records, increase the risk of patients being mistakenly classified as ADHD cases despite exhibiting subthreshold diagnostic symptoms because parental biases and perceptions can influence ADHD status [12]. Likewise, limited sources of information, such as teacher-based reports, only show the child’s behaviour in one setting, with limited insight into functioning in other areas of life [24]. Cultural differences can also affect the robustness of interview methods. This is because some cultures are more tolerant of ADHD symptoms in children and may therefore perceive symptoms as less severe, which leads to underreporting of abnormal signs. For example, Western culture is generally more tolerant of hyperactive symptoms [20]. In contrast, studies which utilise data from national health records rely on more objective and standardised information, which increases generalisability in the findings [12,22].

Medical record data:* *Studies that solely use medical records to identify children with ADHD also pose hindrances in the accuracy of their prevalence estimates because they don’t include children with ADHD symptoms in the community who haven’t been formally diagnosed. This study also doesn’t capture formally diagnosed children who are not in the medical records used by the study for several reasons. For example, some individuals may have been diagnosed in a different country and have not transferred care due to limited access to healthcare, alternative treatment approaches or parents choosing not to pursue an ADHD diagnosis for their children due to perceived stigma surrounding ADHD [21].

Primary care data vs. secondary care data: Furthermore, some studies use limited sources of data, which decreases the accuracy of results. This study used a large nationally representative data set containing primary medical records to calculate ADHD medication use prevalence without accounting for secondary care data. This limits the accuracy of reported prevalence figures because prescriptions for newly diagnosed ADHD patients are under secondary care initially for a time period before prescriptions are transferred to primary care for long-term monitoring. Therefore, even though the majority of ADHD prescriptions are monitored in primary care, excluding secondary care records may be underestimating true ADHD medication use [21].

Study Design

Cross-sectional studies: Cross-sectional studies depict past ADHD prevalence at a specific point in time; however, they are not able to provide insight into factors explaining prevalence trends or variances in prevalence. This is in contrast to longitudinal studies, which show the interaction between prevalence trends over time and associated factors, which helps to contextualise the data by establishing correlative relationships [12].

Longitudinal studies:* *McKechnie et al. (2023) conducted a longitudinal study with a cut-off point in 2018 [21]. Therefore, the study isn’t able to account for the effect of factors which occurred after 2018, hence limiting our ability to interpret the results in the context of 2024. For example, the COVID-19 pandemic significantly reduced access to diagnostic services, which may have an effect on ADHD prevalence rates [21].

Similarly, longitudinal studies are subjected to attrition, which is where participants drop out of the study over time, which may lead to the remaining participants not being representative of the original cohort, especially if there were specific commonalities within the individuals who left the study. For example, those leaving the study were more likely to be from a lower SES [24].

Systematic reviews: Systematic reviews and meta-analyses may potentially miss out high-quality robust studies or have significant heterogeneity across the included studies. This could potentially reduce robustness in the results and our ability to meaningfully interpret them [13,23]. On the other hand, systematic reviews are also able to synthesise high-quality findings by providing a large coverage of the relevant literature. Furthermore, systematic reviews which adhere to strict guidelines (e.g., PRISMA guidelines) and use robust methodological protocols are better able to account for any heterogeneity across studies [23].

Methodological variations: Prevalence trends reported by some studies are subject to misinterpretation when confounding factors, such as SES and adverse childhood experiences, aren’t accounted for in the methodological process. This is because the confounding variable may actually be driving the prevalence trend rather than the suggested factor; hence, we are not able to draw accurate conclusions from the study [12]. Some of the studies have very large sample sizes, which increases the likelihood that the findings of the study are nationally representative and generalisable [10]. Furthermore, even though large studies are more likely to have increased heterogeneity, they are also better equipped to deal with it due to greater statistical power and more rigorous methodological processes, thus resulting in more accurate and robust data [21]. In contrast, Chung et al. (2019) analysed data from one health care plan limited to a single geographical area, which subjects this study to selection bias [22]. This suggests that the data is less likely to be representative of national cohorts and thus decreases our ability to utilise data from the study to compare results with other more generalisable studies [22].

Biases: Studies which have low response rates are vulnerable to response bias, which can skew the findings of the study. The low response rate could introduce response bias and, therefore, introduce an explicit limitation on the generalisability of the reported prevalence data. If there is a systematic difference between the characteristics of non-responders vs. responders, then this would mean that the study cohort is not representative of the population, thereby decreasing the generalisability of the results [24]. For example, if people who were from a higher SES were likely to respond and people from a lower status were less likely to respond, then the study findings would overrepresent higher economic status individuals.

## Conclusions

ADHD has long been recognised as a syndrome which can have debilitating functional effects on those affected. Our understanding of ADHD has undergone many changes as diagnostic criteria have evolved along with our scientific understanding of the condition. Over time, diagnostic criteria have become more inclusive to recognise the broad range of symptomatology and manifestations of ADHD, which has led to an increase in diagnosis rates. Furthermore, increasing awareness amongst underrepresented groups, such as girls and ethnic minorities, has led to more people seeking medical attention for their symptoms. The result is that more people who would previously go undiagnosed are finally being identified and treated for their ADHD. Therefore, although data show an increase in diagnostic ADHD prevalence, in reality, the prevalence of the ADHD phenotype remains relatively constant. ADHD is increasingly becoming labelled as an overdiagnosed disease, which creates stigma and additional barriers for underrepresented individuals seeking diagnostic services. This ultimately leads to undiagnosed ADHD and subsequent disruption in daily functioning. As more people have access to ADHD medication, there are challenges associated with the growing use of stimulants. For example, a major concern of pharmacological ADHD treatment is the poor long-term efficacy and side effects of current medications, which will only become more apparent as stimulants are used more frequently. This highlights the need for increased research and development in ADHD pharmacology from both a public health perspective as well as at an individual patient level.

Social biases amongst individuals, such as teachers and physicians, have also played a role in the societal disparities seen in ADHD prevalence. For example, physicians were less receptive to the concerns of White parents than Black or African American parents, and teachers were more responsive to symptoms in boys than girls. Such biases are not ADHD specific and apply to other mental health conditions in general. This raises the broader issue of the effects of social biases on mental health disparities and the need for public interventions in tackling this issue. For example, training amongst healthcare professionals is essential to ensure they are aware of potential biases that may affect their clinical judgement in the context of their cultural background. It is also essential for physicians to be actively aware of cultural differences and societal norms to enable them to make an accurate diagnosis. In summary, this review showcases the importance of holistically assessing each patient with awareness of potential biases so that professionals can make thoughtful diagnoses on a patient-by-patient basis. A major reason for variation in ADHD prevalence reported by different studies was due to variation in diagnostic methods (e.g., ICD vs. DSM-IV), which highlights the need for increased universality in how ADHD is diagnosed. This is because both overdiagnosis and underdiagnosis lead to increased burden on individuals and their families, as well as fiscal implications for institutions that treat ADHD. Furthermore, this review found that there is no true geographical variation in ADHD prevalence and that it is rather it is due to methodological variation amongst studies. This review has found that ADHD is more prevalent in individuals with a lower socioeconomic status, with rates twice as high in the most deprived areas compared to the least deprived. There is insufficient data regarding the reasoning behind this pattern, which emphasises the need for more research to enable public health institutions to establish interventions to provide suitable support for underprivileged children.

## References

[1] Magnus W, Anilkumar AC, Shaban K (2025). Attention deficit hyperactivity disorder. StatPearls.

[2] Döpfner M, Breuer D, Wille N, Erhart M, Ravens-Sieberer U (2008). How often do children meet ICD-10/DSM-IV criteria of attention deficit-/hyperactivity disorder and hyperkinetic disorder? Parent-based prevalence rates in a national sample--results of the BELLA study. Eur Child Adolesc Psychiatry.

[3] American Psychiatric Association (2012). Diagnostic and Statistical Manual of Mental Disorders, Fifth Edition (DSM-5).

[4] World Health Organization (2004). ICD-10: International Statistical Classification of Diseases and Related Health Problems: Tenth Revision, 2nd Ed. https://iris.who.int/handle/10665/42980.

[5] Harpin VA (2005). The effect of ADHD on the life of an individual, their family, and community from preschool to adult life. Arch Dis Child.

[6] Faraone SV, Biederman J, Mick E (2006). The age-dependent decline of attention deficit hyperactivity disorder: a meta-analysis of follow-up studies. Psychol Med.

[7] Morgan PL, Staff J, Hillemeier MM, Farkas G, Maczuga S (2013). Racial and ethnic disparities in ADHD diagnosis from kindergarten to eighth grade. Pediatrics.

[8] Morgan PL, Hu EH (2023). Sociodemographic disparities in ADHD diagnosis and treatment among U.S. elementary schoolchildren. Psychiatry Res.

[9] Rowland AS, Lesesne CA, Abramowitz AJ (2002). The epidemiology of attention-deficit/hyperactivity disorder (ADHD): a public health view. Ment Retard Dev Disabil Res Rev.

[10] Polanczyk G, de Lima MS, Horta BL, Biederman J, Rohde LA (2007). The worldwide prevalence of ADHD: a systematic review and metaregression analysis. Am J Psychiatry.

[11] Isaksson J, Nilsson KW, Lindblad F (2015). The Pressure-Activation-Stress scale in relation to ADHD and cortisol. Eur Child Adolesc Psychiatry.

[12] Xu G, Strathearn L, Liu B, Yang B, Bao W (2018). Twenty-year trends in diagnosed attention-deficit/hyperactivity disorder among US children and adolescents, 1997-2016. JAMA Netw Open.

[13] Polanczyk GV, Willcutt EG, Salum GA, Kieling C, Rohde LA (2014). ADHD prevalence estimates across three decades: an updated systematic review and meta-regression analysis. Int J Epidemiol.

[14] Abdelnour E, Jansen MO, Gold JA (2022). ADHD diagnostic trends: increased recognition or overdiagnosis?. Mo Med.

[15] Robison LM, Skaer TL, Sclar DA, Galin RS (2002). Is attention deficit hyperactivity disorder increasing among girls in the US? Trends in diagnosis and the prescribing of stimulants. CNS Drugs.

[16] Sclar DA, Robison LM, Bowen KA, Schmidt JM, Castillo LV, Oganov AM (2012). Attention-deficit/hyperactivity disorder among children and adolescents in the United States: trend in diagnosis and use of pharmacotherapy by gender. Clin Pediatr (Phila).

[17] Kazda L, Bell K, Thomas R, McGeechan K, Sims R, Barratt A (2021). Overdiagnosis of attention-deficit/hyperactivity disorder in children and adolescents: a systematic scoping review. JAMA Netw Open.

[18] Massuti R, Moreira-Maia CR, Campani F (2021). Assessing undertreatment and overtreatment/misuse of ADHD medications in children and adolescents across continents: a systematic review and meta-analysis. Neurosci Biobehav Rev.

[19] Martinez-Raga J, Ferreros A, Knecht C, de Alvaro R, Carabal E (2017). Attention-deficit hyperactivity disorder medication use: factors involved in prescribing, safety aspects and outcomes. Ther Adv Drug Saf.

[20] Chan WW, Shum KK, Sonuga-Barke EJ (2022). Attention-deficit/hyperactivity disorder (ADHD) in cultural context: Do parents in Hong Kong and the United Kingdom adopt different thresholds when rating symptoms, and if so why?. Int J Methods Psychiatr Res.

[21] McKechnie DG, O'Nions E, Dunsmuir S, Petersen I (2023). Attention-deficit hyperactivity disorder diagnoses and prescriptions in UK primary care, 2000-2018: population-based cohort study. BJPsych Open.

[22] Chung W, Jiang SF, Paksarian D, Nikolaidis A, Castellanos FX, Merikangas KR, Milham MP (2019). Trends in the prevalence and incidence of attention-deficit/hyperactivity disorder among adults and children of different racial and ethnic groups. JAMA Netw Open.

[23] Ayano G, Demelash S, Gizachew Y, Tsegay L, Alati R (2023). The global prevalence of attention deficit hyperactivity disorder in children and adolescents: an umbrella review of meta-analyses. J Affect Disord.

[24] Rydell M, Lundström S, Gillberg C, Lichtenstein P, Larsson H (2018). Has the attention deficit hyperactivity disorder phenotype become more common in children between 2004 and 2014? Trends over 10 years from a Swedish general population sample. J Child Psychol Psychiatry.

[25] Jin W, Du Y, Zhong X, David C (2014). Prevalence and contributing factors to attention deficit hyperactivity disorder: a study of five- to fifteen-year-old children in Zhabei District, Shanghai. Asia Pac Psychiatry.

[26] Skounti M, Philalithis A, Galanakis E (2007). Variations in prevalence of attention deficit hyperactivity disorder worldwide. Eur J Pediatr.

[27] Rowland AS, Skipper BJ, Rabiner DL, Qeadan F, Campbell RA, Naftel AJ, Umbach DM (2018). Attention-deficit/hyperactivity disorder (ADHD): interaction between socioeconomic status and parental history of ADHD determines prevalence. J Child Psychol Psychiatry.

[28] Madsen KB, Ersbøll AK, Olsen J, Parner E, Obel C (2015). Geographic analysis of the variation in the incidence of ADHD in a country with free access to healthcare: a Danish cohort study. Int J Health Geogr.

[29] Widding-Havneraas T, Markussen S, Elwert F (2023). Geographical variation in ADHD: do diagnoses reflect symptom levels?. Eur Child Adolesc Psychiatry.

[30] Wang T, Liu K, Li Z, Xu Y, Liu Y, Shi W, Chen L (2017). Prevalence of attention deficit/hyperactivity disorder among children and adolescents in China: a systematic review and meta-analysis. BMC Psychiatry.

[31] Akmatov MK, Steffen A, Holstiege J, Hering R, Schulz M, Bätzing J (2018). Trends and regional variations in the administrative prevalence of attention-deficit/hyperactivity disorder among children and adolescents in Germany. Sci Rep.

[32] Posner J, Polanczyk GV, Sonuga-Barke E (2020). Attention-deficit hyperactivity disorder. Lancet.

[33] Matthews M, Nigg JT, Fair DA (2014). Attention deficit hyperactivity disorder. Curr Top Behav Neurosci.

[34] Mahone EM, Denckla MB (2017). Attention-deficit/hyperactivity disorder: a historical neuropsychological perspective. J Int Neuropsychol Soc.

[35] Epstein JN, Loren RE (2013). Changes in the Definition of ADHD in DSM-5: Subtle but Important. Neuropsychiatry (London).

[36] Nøvik TS, Hervas A, Ralston SJ, Dalsgaard S, Rodrigues Pereira R, Lorenzo MJ (2006). Influence of gender on attention-deficit/hyperactivity disorder in Europe--ADORE. Eur Child Adolesc Psychiatry.

[37] Nussbaum NL (2012). ADHD and female specific concerns: a review of the literature and clinical implications. J Atten Disord.

[38] Gershon J (2002). A meta-analytic review of gender differences in ADHD. J Atten Disord.

[39] Quinn PO, Madhoo M (2014). A review of attention-deficit/hyperactivity disorder in women and girls: uncovering this hidden diagnosis. Prim Care Companion CNS Disord.

[40] Slobodin O, Davidovitch M (2019). Gender differences in objective and subjective measures of ADHD among clinic-referred children. Front Hum Neurosci.

[41] Ohan JL, Visser TA (2009). Why is there a gender gap in children presenting for attention deficit/hyperactivity disorder services?. J Clin Child Adolesc Psychol.

[42] Coles EK, Slavec J, Bernstein M, Baroni E (2012). Exploring the gender gap in referrals for children with ADHD and other disruptive behavior disorders. J Atten Disord.

[43] Young S, Adamo N, Ásgeirsdóttir BB (2020). Females with ADHD: an expert consensus statement taking a lifespan approach providing guidance for the identification and treatment of attention-deficit/ hyperactivity disorder in girls and women. BMC Psychiatry.

[44] Coker TR, Elliott MN, Toomey SL (2016). Racial and ethnic disparities in ADHD diagnosis and treatment. Pediatrics.

[45] Fadus MC, Ginsburg KR, Sobowale K, Halliday-Boykins CA, Bryant BE, Gray KM, Squeglia LM (2020). Unconscious bias and the diagnosis of disruptive behavior disorders and ADHD in African American and Hispanic youth. Acad Psychiatry.

[46] Leslie LK, Plemmons D, Monn AR, Palinkas LA (2007). Investigating ADHD treatment trajectories: listening to families' stories about medication use. J Dev Behav Pediatr.

[47] Crapanzano KA, Deweese S, Pham D, Le T, Hammarlund R (2023). The role of bias in clinical decision-making of people with serious mental illness and medical co-morbidities: a scoping review. J Behav Health Serv Res.

[48] Fairman KA, Peckham AM, Sclar DA (2020). Diagnosis and treatment of ADHD in the United States: update by gender and race. J Atten Disord.

[49] Collins KP, Cleary SD (2016). Racial and ethnic disparities in parent-reported diagnosis of ADHD: National Survey of Children's Health (2003, 2007, and 2011). J Clin Psychiatry.

[50] Yeung A, Ng E, Abi-Jaoude E (2022). TikTok and attention-deficit/hyperactivity disorder: a cross-sectional study of social media content quality. Can J Psychiatry.

[51] Moffitt TE, Melchior M (2007). Why does the worldwide prevalence of childhood attention deficit hyperactivity disorder matter?. Am J Psychiatry.

[52] Tripp G, Luk SL, Schaughency EA, Singh R (1999). DSM-IV and ICD-10: a comparison of the correlates of ADHD and hyperkinetic disorder. J Am Acad Child Adolesc Psychiatry.

[53] Buitelaar JK, Barton J, Danckaerts M (2006). A comparison of North American versus non-North American ADHD study populations. Eur Child Adolesc Psychiatry.

[54] Matza LS, Paramore C, Prasad M (2005). A review of the economic burden of ADHD. Cost Eff Resour Alloc.

[55] Russell AE, Ford T, Williams R, Russell G (2016). The association between socioeconomic disadvantage and attention deficit/hyperactivity disorder (ADHD): a systematic review. Child Psychiatry Hum Dev.

[56] Russell AE, Ford T, Russell G (2015). Socioeconomic associations with ADHD: findings from a mediation analysis. PLoS One.

[57] Kvist AP, Nielsen HS, Simonsen M (2013). The importance of children's ADHD for parents' relationship stability and labor supply. Soc Sci Med.

[58] Russell G, Ford T, Rosenberg R, Kelly S (2014). The association of attention deficit hyperactivity disorder with socioeconomic disadvantage: alternative explanations and evidence. J Child Psychol Psychiatry.

[59] Biederman J, Faraone SV, Spencer TJ, Mick E, Monuteaux MC, Aleardi M (2006). Functional impairments in adults with self-reports of diagnosed ADHD: a controlled study of 1001 adults in the community. J Clin Psychiatry.

[60] Jangmo A, Stålhandske A, Chang Z (2019). Attention-deficit/hyperactivity disorder, school performance, and effect of medication. J Am Acad Child Adolesc Psychiatry.

[61] Leitch S, Sciberras E, Post B, Gerner B, Rinehart N, Nicholson JM, Evans S (2019). Experience of stress in parents of children with ADHD: a qualitative study. Int J Qual Stud Health Well-being.

[62] Wilens TE, Martelon M, Joshi G, Bateman C, Fried R, Petty C, Biederman J (2011). Does ADHD predict substance-use disorders? A 10-year follow-up study of young adults with ADHD. J Am Acad Child Adolesc Psychiatry.

[63] Murphy K, Barkley RA. (1996). Attention deficit hyperactivity disorder adults: comorbidities and adaptive impairments. Compr Psychiatry.

[64] Sibley MH, Swanson JM, Arnold LE (2017). Defining ADHD symptom persistence in adulthood: optimizing sensitivity and specificity. J Child Psychol Psychiatry.

[65] Faraone SV, Larsson H (2019). Genetics of attention deficit hyperactivity disorder. Mol Psychiatry.

[66] Schachter HM, Pham B, King J, Langford S, Moher D (2001). How efficacious and safe is short-acting methylphenidate for the treatment of attention-deficit disorder in children and adolescents? A meta-analysis. CMAJ.

[67] Greenhill LL, Swanson JM, Hechtman L (2020). Trajectories of growth associated with long-term stimulant medication in the multimodal treatment study of attention-deficit/hyperactivity disorder. J Am Acad Child Adolesc Psychiatry.

[68] Faraone SV, Biederman J, Morley CP, Spencer TJ (2008). Effect of stimulants on height and weight: a review of the literature. J Am Acad Child Adolesc Psychiatry.

[69] Greenhill L, Kollins S, Abikoff H (2006). Efficacy and safety of immediate-release methylphenidate treatment for preschoolers with ADHD. J Am Acad Child Adolesc Psychiatry.

[70] Ibrahim K, Donyai P (2015). Drug holidays from ADHD medication: international experience over the past four decades. J Atten Disord.

[71] Chang Z, Lichtenstein P, Halldner L (2014). Stimulant ADHD medication and risk for substance abuse. J Child Psychol Psychiatry.

[72] DeSantis AD, Anthony KE, Cohen EL (2013). Illegal college ADHD stimulant distributors: characteristics and potential areas of intervention. Subst Use Misuse.

[73] Wilens TE, Adler LA, Adams J (2008). Misuse and diversion of stimulants prescribed for ADHD: a systematic review of the literature. J Am Acad Child Adolesc Psychiatry.

[74] Cottler LB, Striley CW, Lasopa SO (2013). Assessing prescription stimulant use, misuse, and diversion among youth 10-18 years of age. Curr Opin Psychiatry.

[75] Poulin C (2001). Medical and nonmedical stimulant use among adolescents: from sanctioned to unsanctioned use. Can Med Assoc J.

[76] Rabiner DL (2013). Stimulant prescription cautions: addressing misuse, diversion and malingering. Curr Psychiatry Rep.

[77] Cooper WO, Habel LA, Sox CM (2011). ADHD drugs and serious cardiovascular events in children and young adults. N Engl J Med.

[78] Barbaresi WJ, Katusic SK, Colligan RC, Weaver AL, Jacobsen SJ (2007). Long-term school outcomes for children with attention-deficit/hyperactivity disorder: a population-based perspective. J Dev Behav Pediatr.

[79] Kortekaas-Rijlaarsdam AF, Luman M, Sonuga-Barke E, Oosterlaan J (2019). Does methylphenidate improve academic performance? A systematic review and meta-analysis. Eur Child Adolesc Psychiatry.

[80] Lichtenstein P, Halldner L, Zetterqvist J (2012). Medication for attention deficit-hyperactivity disorder and criminality. N Engl J Med.

[81] Chang Z, Quinn PD, Hur K, Gibbons RD, Sjölander A, Larsson H, D'Onofrio BM (2017). Association between medication use for attention-deficit/hyperactivity disorder and risk of motor vehicle crashes. JAMA Psychiatry.

[82] Greydanus DE, Nazeer A, Patel DR (2009). Psychopharmacology of ADHD in pediatrics: current advances and issues. Neuropsychiatr Dis Treat.

[83] Charach A, Fernandez R (2013). Enhancing ADHD medication adherence: challenges and opportunities. Curr Psychiatry Rep.

